# MSDESIS: Multi-task stereo disparity estimation and surgical instrument segmentation

**DOI:** 10.1109/TMI.2022.3181229

**Published:** 2022-06-08

**Authors:** Dimitrios Psychogyios, Evangelos Mazomenos, Francisco Vasconcelos, Danail Stoyanov

**Affiliations:** Wellcome EPSRC Center for Interventional and Surgical Sciences (WEISS), University College London, London WC1E 6BT, U.K.

**Keywords:** Computer assisted interventions, Computational stereo, Surgical vision, Instrument segmentation

## Abstract

Reconstructing the 3D geometry of the surgical site and detecting instruments within it are important tasks for surgical navigation systems and robotic surgery automation. Traditional approaches treat each problem in isolation and do not account for the intrinsic relationship between segmentation and stereo matching. In this paper, we present a learning-based framework that jointly estimates disparity and binary tool segmentation masks. The core component of our architecture is a shared feature encoder which allows strong interaction between the aforementioned tasks. Experimentally, we train two variants of our network with different capacities and explore different training schemes including both multi-task and single-task learning. Our results show that supervising the segmentation task improves our network’s disparity estimation accuracy. We demonstrate a domain adaptation scheme where we supervise the segmentation task with monocular data and achieve domain adaptation of the adjacent disparity task, reducing disparity End-Point-Error and depth mean absolute error by 77.73% and 61.73% respectively compared to the pre-trained baseline model. Our best overall multi-task model, trained with both disparity and segmentation data in subsequent phases, achieves 89.15% mean Intersection-over-Union in RIS and 3.18 millimetre depth mean absolute error in SCARED test sets. Our proposed multi-task architecture is real-time, able to process (1280x1024) stereo input and simultaneously estimate disparity maps and segmentation masks at 22 frames per second. The model code and pre-trained models are made available: https://github.com/dimitrisPs/msdesis

## I Introduction

Stereo endoscopes are increasingly used in minimally invasive surgery with both robotic and manual approaches as the main sensory signal to enable three-dimensional (3D) visualization of the surgical site and depth perception. The visual signal from the scope can be used for computational inference to underpin computer assisted interventions (CAI) ranging from post-operative analytics of surgical workflow [2], intra-operative overlaying pre-operative data onto the surgeon’s field of view [3] all the way through to active robotic motion compensation [4]. Such CAI systems require solutions to multiple vision problems for understanding the 3D geometry of the surgical site and, at the same time, the location of anatomy and instruments as well as their motion and deformation over time. Such vision artificial intelligence systems are typically developed and operate as standalone modules but there could be important potential advantages of combining them in a multi-task fashion which may include more efficient utilization of computational resources as well as performance increases of at least one of the inference tasks [5], [6]. Yet the benefits of joint or multi-task solutions have largely been unexplored so far in CAI applications and applying multitask learning in surgical video has also been inhibited by a lack of relevant datasets to enable model development.

Standalone surgical tool detection and segmentation models typically incorporate pre-trained feature extraction layers [7] and have rapidly improved in performance with increasing labelled data availability and the use of simulation for data generation [8], [9]. Meanwhile, 3D geometry estimation models from stereo have seen less active development but recent additions to the available datasets for training and validation may facilitate learning-based disparity estimation [10]. Furthermore, self-supervised disparity estimation approaches have been developed and aim to alleviate the need for depth ground truth data [11], [12], [13]. Related approaches also focus on the development of both camera motion estimation and 3D reconstruction [14], [15] and on the estimation of depth using monocular endoscopes [16]. The amount of recently proposed self-supervised approaches to solve disparity and depth estimation highlights the data scarcity problem present in CAI. All of the aforementioned developments could benefit from exploring solving the problems of geometry estimation and semantic segmentation in a joint model, and leveraging existing segmentation datasets to improve depth and disparity estimation accuracy.

**Fig. 1:**
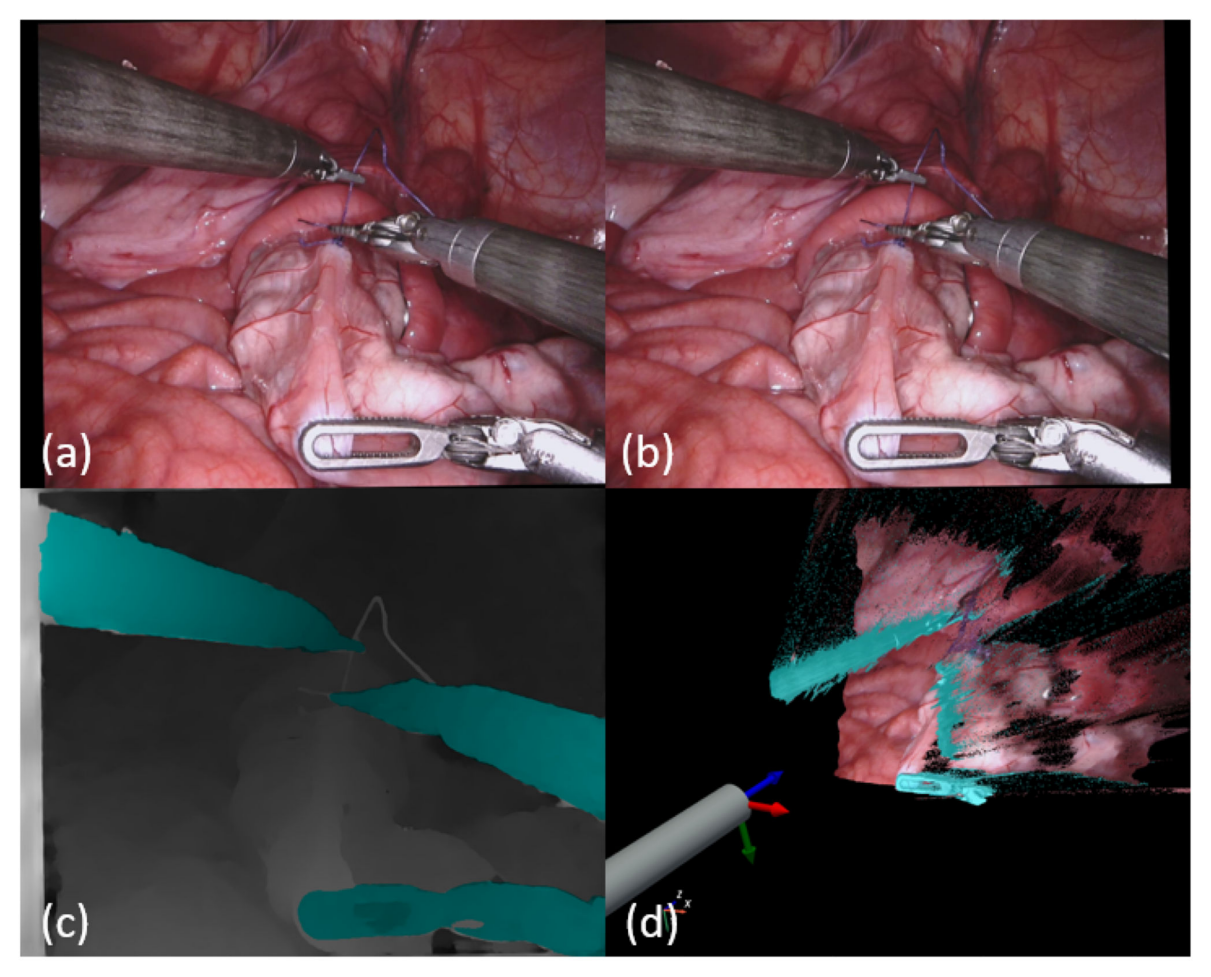
Left (a) and right (b) stereo rectified endoscopic frame form the RIS dataset [1]. The combined output (c) of our multi-task framework, fine-tuned with monocular segmentation data, simultaneously predicting disparity and binary surgical tool segmentation masks (cyan) in the left rectified frame of reference. Rendition (d) showing the endoscope and the combined output of the network reconstructed in 3D.

In this paper, we propose a multi-task framework for the joint estimation of disparity and binary tool segmentation masks as illustrated in Fig. 1. Our modular architecture consists of a shared feature extraction encoder between a disparity and a segmentation head. We use modified versions of MADNet [17] and U-Net [18] for the disparity and segmentation tasks respectively that allow our architecture to be fast, enabling its use in real-time CAI systems. We evaluate our multi-task approach for segmentation accuracy on the RIS dataset [1] and we measure depth and disparity error on the recent SCARED dataset [10].

Through a series of experiments, we investigate the benefits of multi-task learning and propose solutions to mitigate the disparity data scarcity problem present in CAI. Starting from a disparity pre-trained network, our results show that supervising an adjacent segmentation task leads to adaptation of the common feature encoder in the surgical domain which in turn increases the performance of the disparity task. This is a significant finding, as it allows us to use a lot easier to generate, monocular segmentation data to effectively adapt our disparity networks.

Our main contributions are the following:

- Develop the first multi-task framework for joint estimation of disparity and tool segmentation able to run at real-time.
- Demonstrate a technique for disparity domain adaptation, without stereo ground truth, using monocular segmentation data.
- Evaluate different training schemes to achieve disparity estimation and tool segmentation multi-tasking using uni-modal datasets.
- Open source our network model^1^ and data processing Code^2 3^

## II Related Work

### A Disparity Estimation in Surgery

In CAI, until recently data scarcity limited stereo-based 3D reconstruction to handcrafted approaches [19], [20], [21] with datasets [22], [23], [24] designed for validation. Despite promising unsupervised disparity estimation methods [25], [26], [27] and methods attempting domain transfer from nonsurgical training data [28] practical performance deteriorates in surgical scenes. The SCARED [10] challenge at EndoVis’19 presented the first dataset aimed to train supervised deep learning methods which dominate non-surgical vision applications. The winning team used a PSMNet [29] with a spatial pyramid module to enlarge the receptive field of its feature vectors and hourglass module for disparity refinement, achieving depth mean absolute error (MAE) of 3.54 millimetre (mm). Subsequently, using DeepPrunner [30] reduced this error to 2.34mm. Yet, these approaches use 3D convolutions in the disparity aggregation phase, hindering their run-time performance. In this paper, we build upon the fast and lightweight MADNet [17] architecture, by adding residual disparity refinement in every disparity scale and a two-dimensional (2D) hourglass disparity refinement module in the output stage. This lightweight architecture enables us to process 1280x1024 stereo images at 22 frames per second (FPS) while multi-tasking.

### B Multi-task Disparity Learning

Intuitively, if multiple models share a single feature extraction backbone, the inference time and memory requirements of the whole system decrease significantly, as features need to be extracted only once. In addition, multi-task networks can increase the performance of at least a single task with examples in semantic segmentation to increase its disparity estimation [6] and edge detection sub-networks to improve the stereo estimation performance near object boundaries [5]. Recently, highly multi-tasking models like SENSE [31], can simultaneously predict segmentation masks, occlusion masks, optical flow, and disparity. Because all three tasks share a common feature encoder, SENSE can run in real-time and this is important for CAI. However, the disparity and segmentation datasets in surgery are vastly dissimilar to those in the general literature, and datasets including both segmentation and disparity annotations do not yet exist for laparoscopy and existing approaches cannot be applied directly in CAI. Because of the data scarcity in the surgical domain, we focus on the domain adaptation aspect of multi-tasking rather than optimizing the accuracy of our network. In our approach, we first pre-train out method under full disparity supervision on a synthetic disparity dataset and then experiment with various training workflows, including multi-task learning as well as changing the supervised task in subsequent training phases.

### C Instrument Segmentation

The release of labelled tool datasets [1], [32] has enabled deep learning approaches to be used for tool segmentation. Starting from a pre-trained encoder, TernausNet [7] was fine-tuned for the RIS challenge [1], and set the state-of-the-art binary tool segmentation accuracy at 88.8% mean Intersection-over-Union (mIoU). Toolnet [33], an architecture for tool segmentation focused on run-time performance, achieved 78.5% mIoU in the same challenge while being able to process (720×576) input at 29 FPS. More recently, ST-MTL [34] was developed to jointly estimate surgical instrument segmentation and saliency detection, setting the current state-of-the-art in [1] at 91% mIoU. In our work, we designed a segmentation head inspired by U-net, which is compatible with the pyramidal structure of our feature encoders. In segmentation-only mode, our lightweight architecture can process monocular (1280x1024) input at over 40 FPS on an Nvidia Tesla V100. Our best overall lightweight network achieves binary segmentation performance of 89.15% mIoU while our ResNet34 segmentation optimized variants reach 90.46% mIoU.

## III Method

### A Network Architecture

**Fig. 2:**
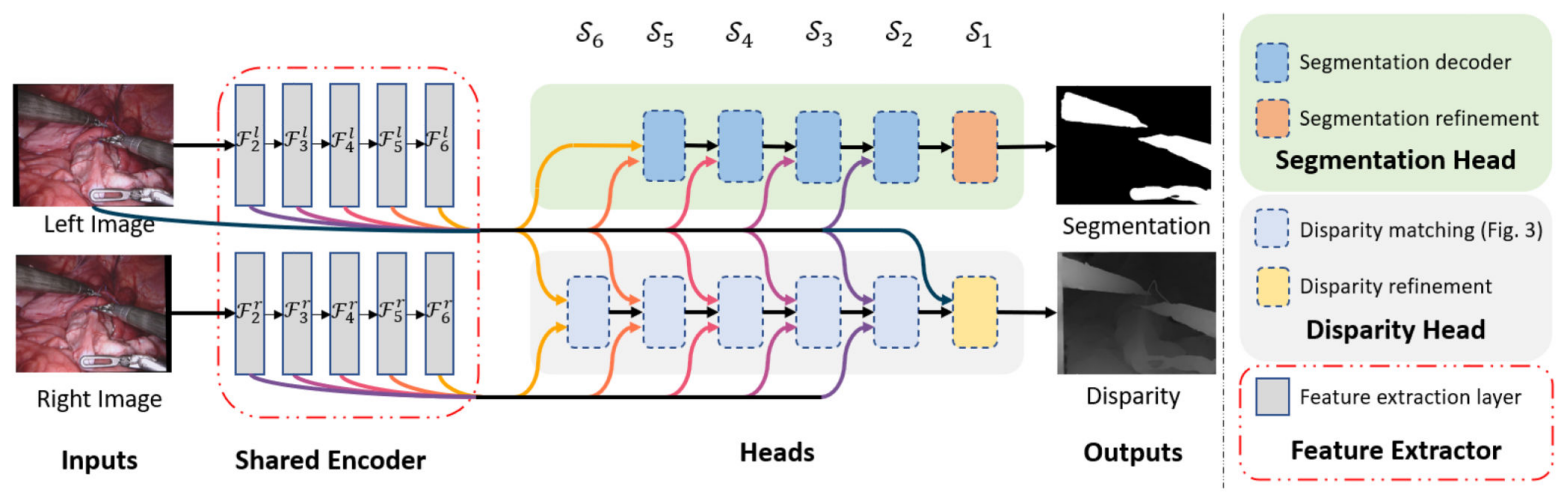
The architecture of our multi-task framework with both heads attached. Left and right rectified images are processed by the shared feature encoder which outputs feature maps *𝓕_i_* at different scales *𝒮_i_*. Those features are inputs to each task-specific sub-network(head) that make the final predictions. The modular design allows us to interchange or remove parts of the network.

Fig.2 shows a diagram of our modular architecture. The proposed framework is built upon a single convolutional encoder that extracts deep feature representation from input images, at 5 different scales *S_i_*, *i* ∈ (2, 3, 4, 5, 6). Each task has a dedicated convolutional sub-network (head) that makes predictions based on multi-scale inputs from the common feature encoder. We test two variations of the common feature encoder because we want to investigate how its capacity affects the performance of our networks, especially in the multitask setting. The two backbones produce features at the same scales but different feature lengths, meaning that we need to have different versions of heads for each encoder. Our segmentation head is a variation of U-Net [18], outputting binary segmentation masks at the same resolution as the inputs. Our disparity regression head follows a 2D cascade cost volume design which helps to keep our disparity head’s memory requirements and execution time low. Since disparity is inversely proportional to depth, to make sure disparity prediction of tools close to the endoscope is possible, our disparity head is configured to regress disparity values up to 320 pixels.

The interaction between the two sub-networks is limited to the common feature extraction encoder, giving us the flexibility to completely remove the head of one task without affecting the performance of the other. The advantages of this modular design are identifiable during both training and inference. During training, we can use monocular segmentation data to fine-tune the segmentation head and the shared feature encoder, which leads to the adaption of the disparity task to new domains without the need for perfectly calibrated stereo input. During inference, if one of the two tasks is not required, the network can work in single-task mode with increased run-time performance and a smaller memory footprint.

#### 1) Shared Feature Encoder

Our ResNet variant uses a standard ResNet34 with batch normalization layers, whereas our lightweight network adopts a modified version of the pyramidal feature encoder from MADNet. Both feature encoders output features maps at 5 different scales 𝓕_*i*_, *i* ∈ (2, 3, 4, 5, 6). At each scale, 𝓕_*i*_ has half the resolution of 𝓕_*i*−1_ but longer feature vectors. The resolution of a feature map at scale *S_i_* is 2^−(*i*−1)^ and the length of the feature vectors is 16,32,64,96,128 and 64,64,128,256,512 for the lightweight and the ResNet34 encoders respectively.

The MADNet encoder is modified in two ways. First, we omit the last output scale at /64 resolution and second add batch normalization layers between the activation functions and the convolutional layers. The resulting encoder consists of 5 down-sampling blocks of two 3x3 2D convolutions, each followed by a batch normalization layer and a LeakyReLU activation function with a negative slope of 0.1. At each block, the first convolution is used for halving the size of the input tensor, using a strided convolution of 2, and increasing the length of the feature map. The second further processes the feature maps without affecting their dimensions. The lightweight encoder has 0.5 million parameters which is much less compared to the 21 million parameters of the ResNet34 encoder. The lower parameter count in addition to the short length feature output contributes greatly to the fast inference times of our lightweight variant as we discuss in section VI-F.

#### 2) Disparity Head

**Fig. 3:**
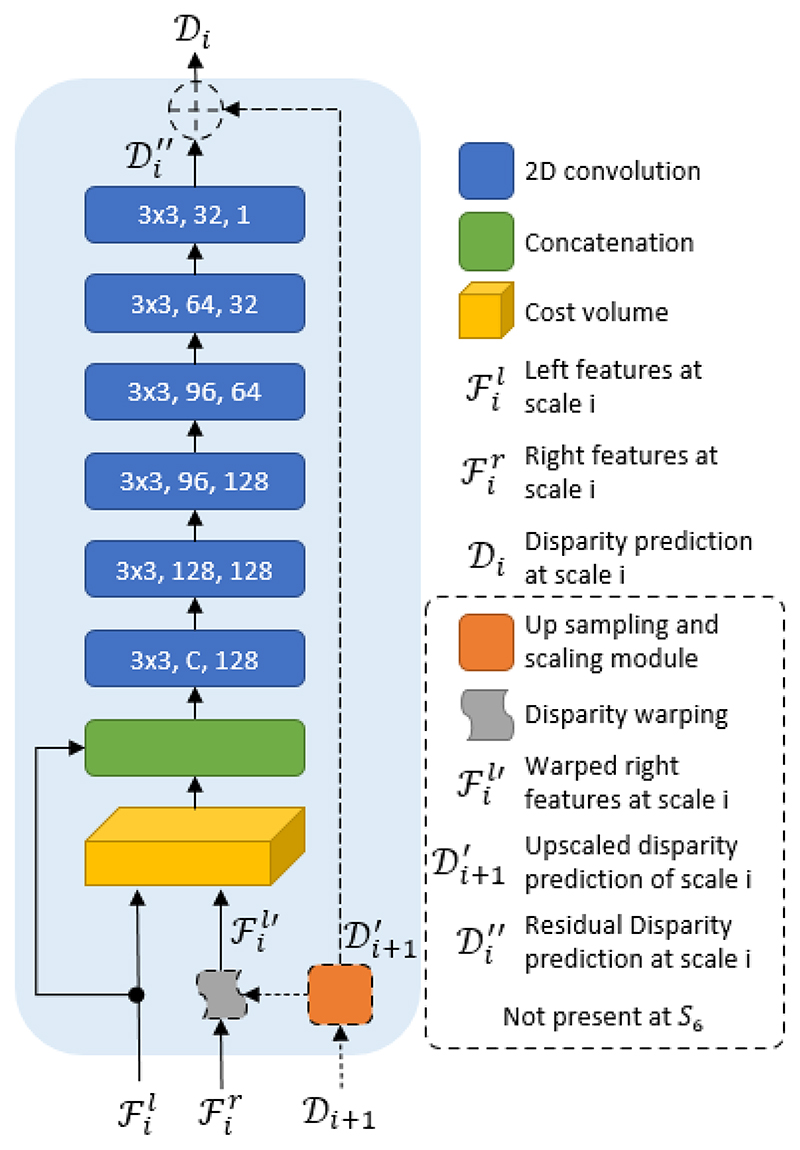
The internal structure of the disparity matching module. Right features ${ℱ}_{i}^{r}$ are warped based on the up-sampled and upscaled disparity estimation of a previous scale $D_{i+1}^{\prime }$, forming ${ℱ}_{i}^{l^{\prime }}$ which, together with the left features ${ℱ}_{i}^{l}$, build a shallow cost volume. This cost volume and ${ℱ}_{i}^{l}$ are concatenated and processed by a series of convolution layers to create a residual disparity connection. The dotted modules and connections are absent in *S*_6_ because our network does not predict disparity at *S*_7_

Our disparity head estimates and refines disparity hierarchically. It consists of cascaded cost volume construction and refinement modules at *S*_2,3,4,5,6_ scales and a disparity refinement module at full resolution *S*_1_. Following Fig.3, let ${ℱ}_{i}^{l}$ and ${ℱ}_{i}^{r}$ be the left and right features maps, respectively, at scale *S_i_*. We exploit the up-scaled and upsampled disparity output of the previous scale, $D_{i+1}^{\prime }$, to prealign ${ℱ}_{i}^{r}$ to ${ℱ}_{i}^{l^{\prime }}$ and construct a bidirectional shallow cost volume with maximum disparity search range of ±2. To get $D_{i+1}^{\prime }$ we have to up-sample and up-scale 𝒟_*i*+1_ by a factor 2. The up-sampling is done using bi-linear interpolation, while the up-scaling is learned and applied by a single convolutional layer. In addition to scaling, the convolutions layer refines the up-sampled disparity by reducing interpolation artifacts. The shallow cost volume and ${ℱ}_{i}^{l}$ are concatenated together and passed into a series of convolutional layers which perform both refinement and aggregation. The last of those convolutional layers regresses the residual disparity $D_{i}^{\prime \prime }$. The final output of this layer is $D_{i}=D_{i}^{\prime \prime }+D_{i+1}^{\prime }$

At *S*_6_ we do not have a disparity estimate from a previous scale, hence feature pre-alignment is not possible. Disparity matching at this scale can be visualized by removing the dashed lines and layers in Fig. 3. We construct a unidirectional cost volume directly from ${ℱ}_{6}^{l}$ and ${ℱ}_{6}^{r}$ and the rest of the process is the same, except now we do not have a disparity identity connection. We configure the maximum disparity search space to be 320 pixels. At *S*_6_, the disparity search range of the cost volume is equal to the maximum desired disparity divided by the maximum scale factor(32) plus one plane for the zero displacement, therefore the depth of the cost volume at this scale is $\frac{320}{32}+1=11$. Unlike traditional disparity estimation networks [29], [35], the cascade cost volume design we adopt, allows us to configure a large disparity search space without a significant drop in run-time performance or increase in memory requirements.

At full resolution, instead of constructing a cost volume, we up-scale 𝒟_2_ and refine it using a 2D hourglass module [29] as a residual connection. This is done for the following 2 reasons: i) We do not have feature outputs at full resolutions and the alternative of constructing a cost volume from raw pixels values, produces noisy disparity outputs. ii) Disparity matching networks like ours assume accurate stereo rectified input frames and inaccuracies in calibration parameters may result in stereo rectifications that exhibit a small vertical offset between corresponding scan-lines. Avoiding cost volume construction at *S*_1_ enables the disparity head to have a small tolerance of about 2 pixels in vertical feature misalignment between stereo views. All the convolutional layers, except those that regress disparity, are followed by a batch normalization layer and a LeakyReLu activation function with a negative slope of 0.1. To keep the magnitude of forward-pass values small, when building the cost volume, instead of computing the dot product between the ${ℱ}_{i}^{l}$ and ${ℱ}_{i}^{l^{\prime }}$ we multiply the feature maps element-wise and compute the mean across the feature dimension. We do this because forward passes with the ResNet backbone resulted in values that caused overflow in half-precision and in our pipelines we were using mixed precision.

#### 3) Segmentation Head

Our segmentation sub-network follows the U-Net architecture with its final layer exporting binary tool segmentation masks at full resolution. Because our feature encoder outputs features at 5 scales starting from /2 resolution and down to /32, our segmentation head uses skip connections up to half resolution. To get prediction at *S*_1_, we up-sample the output of *S*_2_ and convolve it to get the final segmentation logits. Up-sampling within the segmentation head is done using 4x4 2D transposed convolutions with stride of 2, ensuring that up-sampled features match the dimensions of the skip connections. The concatenated features from the encoder and the up-sampled features from the previous scale, get processed by 2 3x3 convolutions followed by a batch normalization and a LeakyReLu activation function(0.1 negative slope), each. During inference, since we only make binary predictions, the output logits are fed into a sigmoid activation function which maps the output range between 0 and 1. Experiments in our evaluation set showed that a segmentation threshold of 0.5 provides optimal results, thus this value is used across all reported results and experiments.

### B Training Modes

#### 1) Pre-training

We pre-train our shared feature encoder and disparity head on the FlyingThings3D dataset [35]. Since there is not any segmentation signal during this phase, the layers of the segmentation head are absent to reduce the graphics processing unit (GPU) memory footprint and training time. We fully supervise disparity predictions in all scales *S_i_*, *i* ∈ [1, 6]. Since only the disparity output of *S*_1_ has the same resolution as the ground truth, to compute the loss for lower scales we first bi-linearly up-sample and upscale the intermediate outputs to the resolution and scale of the ground truth. Additionally, we ignore pixels whose values are more than 320 pixels because they exceed the max disparity search range of our disparity head. At each scale *S_i_* we compute the disparity loss 𝓛*_disp,i_* (1), using the smooth L1 function as defined in (2), where *N* is the total number of valid pixels, *d* is the ground truth disparity, and ${\hat{d}}_{i}$ is the disparity prediction at scale *S_i_*. The final loss is the mean loss across all scales.

(1) $${ℒ}_{\text{disp},i}\left(d,{\hat{d}}_{i}\right)=\frac{1}{N}\sum_{j=1}^{N}smooth_{-}\text{L}1\left(d_{j}-\hat{d_{ij}}\right)$$

where,

(2) $$\text{smooth\_L1 }(w)=\{\begin{array}{ll} 0.5{\left(w\right)}^{2}, & \text{ if }|w|<1\\ |w|-0.5, & \text{ otherwise } \end{array}$$

#### 2) Multi-task learning

During this phase we aim to train the segmentation task and also adapt the pre-trained disparity layers to the surgical domain, effectively achieving multi-tasking. In order to train our architecture jointly and end-to-end, we construct and use a stereo rectified version of the Robotics Instrument Segmentation(RIS) dataset from MICCAI 2017 EndoVis challenge [1] which offers tool segmentation masks in the left frame of a stereo image pair. Details about the dataset construction can be found in section IV-B.1. The ground truth labels are suitable for fully supervising the segmentation task, while the stereo rectified images allow for disparity self-supervision. We only self-supervise the disparity output at *S*_1_ because we experimentally found that self-supervising disparity across all scales does not result in significant gains in accuracy and is much slower. The multi-task loss 𝓛_mt_ (3), used during this phase, is a weighted sum of the segmentation loss term 𝓛_seg_ (4) and the self-supervision disparity loss term 𝓛_ss−disp_ (7).

(3) $${ℒ}_{\text{mt}}=\alpha_{\text{mt}}{ℒ}_{\text{ss}-disp}\left(I^{l},I^{r},\hat{d}\right)+\left(1-\alpha_{\text{mt}}\right){ℒ}_{\text{seg}}(s,\hat{s})$$

𝓛_seg_ (4) is a combination of the weighted binary cross entropy loss 𝓛_WBCE_ (5) and soft dice loss 𝓛_DC_ (6).

(4) $${ℒ}_{\text{seg}}(s,\hat{s})=0.5*{ℒ}_{\text{WBCE}}(s,\hat{s})+0.5*{ℒ}_{\text{DC}}(s,\hat{s})$$

(5) $${ℒ}_{\text{WBCE}}(s,\hat{s})=-\sum_{j=1}^{N}\left(\beta *s_{j}\log\left({\hat{s}}_{j}\right)+\left(1-s_{j}\right)\log\left(1-{\hat{s}}_{j}\right)\right)$$

(6) $${ℒ}_{\text{DC}}(s,\hat{s})=1-\frac{2\sum_{j=1}^{N}s_{j}{\hat{s}}_{j}+\epsilon }{\sum_{j=1}^{N}s_{j}+\sum_{j=1}^{N}{\hat{s}}_{j}+\epsilon }$$

In the above equations, *s* is the reference segmentation mask, *ŝ* is the segmentation prediction of our network passed through a sigmoid activation function. In (6), *ϵ* = 1e − 5 was added for numerical stability. 𝓛_WBCE_ (5) is used to help with class imbalance during training and 𝓛_DC_ (6) improves segmentation performance near tool edges.

𝓛_ss−disp_ (7) is a combination of the photometric loss 𝓛_ph_ (8), the structural similarity image metric loss 𝓛_ssim_ (9), and the disparity smoothness loss 𝓛_smooth_ (11).

(7) $$\begin{array}{c} {ℒ}_{\text{ss}-disp}\left(I^{l},I^{r},\hat{d}\right)=\\ \beta_{\text{ss}}\left(\alpha_{\text{ss}}{ℒ}_{\text{ph}}\left(I^{l},I^{l^{\prime }}\right)+\left(1-\alpha_{\text{ss}}\right){ℒ}_{\text{s}sim}\left(I^{l},I^{l^{\prime }}\right)\right)\\ +\left(1-\beta_{\text{ss}}\right){ℒ}_{\text{smooth}}\left(I^{l},\hat{d}\right) \end{array}$$

𝓛_ph_ (8) is defined as the smooth L1 loss (2) between the left input image *I^l^* and the right input image, warped to the left *I^l′^* based on the disparity output $\hat{d}$ of the network. Because we are using a fully differentiable warping module [36], the gradient flows from *I^l′^* to *d*, enabling training of the disparity head. We do not handle occlusions while image warping, meaning that even if the disparity prediction corresponds to correct depth, *I^l′^* will not resemble *I^l^* in occluded regions.

(8) $${ℒ}_{\text{ph}}\left(I^{l},I^{l^{\prime }}\right)=\frac{1}{N}\sum_{j=1}^{N}smooth\_L1\left(I_{j}^{l}-I_{j}^{l^{\prime }}\right)$$

𝓛_ssim_ (9) is a loss term based on the structural similarity image metric SSIM (10). In our implementation, (10) is computed on 11x11 image patches between the *I^l^* and *I^l′^* and then information is averaged resulting in the SSIM_m_(*I^l^, I^l′^*) of Eq. (9).

(9) $${ℒ}_{\text{ssim}}\left(I^{l},I^{l^{\prime }}\right)=\frac{1-SSIM_{\text{m}}\left(I^{l},I^{l^{\prime }}\right)}{2}$$

(10) $$\begin{array}{c} SSIM(u,v)=\frac{\left(2\mu_{u}\mu_{v}+c1\right)\left(2\sigma_{uv}+c2\right)}{\left(\mu_{u}^{2}+\mu_{v}^{2}+c1\right)\left(\sigma_{u}^{2}+\sigma_{v}^{2}+c_{2}\right)}\\ c1=0.01^{2},c2=0.03^{2} \end{array}$$

In (10), $\sigma_{u}^{2}$, *μ_u_*, *σ_uv_* indicate respectively, the variance of input *u*, the mean of input *u* and the covariance between inputs *u* and *v*.

𝓛_smooth_ (11) penalizes sharp disparity transitions in the absence of edges in *I^l^* using an edge-aware disparity smoothness term.

(11) $$\begin{array}{ll} {ℒ}_{\text{smooth }}\left(I^{l},\hat{d}\right) & =\frac{1}{N}\sum_{j=1}^{N}\left|\partial_{x}{\hat{d}}_{j}\right|e^{-\|\partial_{x}I_{j}^{l}\|}\\ \; & +\frac{1}{N}\sum_{j=1}^{N}\left|\partial_{y}{\hat{d}}_{j}\right|e^{-\|\partial_{y}I_{j}^{l}\|} \end{array}$$

In 𝓛_smooth_ (11), |·| computes the absolute value and ||·|| computes the mean across the 3 colour channels. Additionally, while computing (11), we normalize $\hat{d}$ by dividing its values with the max disparity search range (320). This is done to bring *d* in the same scale as *I^l^* and by extension 𝓛_smooth_ (11) at the same scale as the rest of the loss terms in 𝓛_ss−disp_ (7). Having all terms of (7) at the same scale, facilitated our search for good *α*_ss_ and *β*_ss_ coefficients during development.

## IV Datasets and Pre-Processing

### A Datasets

During this work, we used the following datasets.

#### 1) FlyingThings3D

The clean pass version FlyingThings3D [35] dataset was used to pre-train the disparity sub-network. This dataset consists of 21818 training and 4248 evaluation synthetically generated stereo samples, with a resolution of (960x540) and dense disparity annotation. The size of this dataset makes it possible to train disparity estimation networks without over-fitting.

#### 2) Robotics Instrument Segmentation Dataset

The segmentation dataset used in this work was from the MICCAI 2017 Robotic Instrument Segmentation Challenge [1]. This dataset was generated from 10 abdominal porcine procedures, recorded using daVinci Xi systems. For each procedure, 300 (1280x1024) stereo frames were selected and tool annotations were created. Ground truth segmentation was provided for the first 225 frames of the initial 8 sequences and withheld for the rest for evaluation purposes.

#### 3) SCARED dataset

We train and assess the disparity performance of our model, in the SCARED dataset [10]. SCARED provides 7 training and 2 test videos of different porcine cadavers, acquired using a da Vinci Xi surgical system. Ground truth information was captured directly into the camera’s frame of reference using structured light, completely removing the registration errors between reference depth information and RGB images. The resolution of the videos is (1280x1024) and the ground truth is provided in the form of 3-channel depth images where each channel corresponds to a dimension in the Cartesian space.

### B Data Pre-processing

#### 1) Robotics Instrument Segmentation Dataset

The RIS [1] dataset provides both left and right image frames along with stereo calibration parameters. In some of our experiments, we rely on a stereo rectified version of RIS, however, using the provided calibration parameters to stereo rectify the red-green-blue (RGB) frames and segmentation masks is not trivial. The images provided with RIS were captured using a daVinci Xi at (1280x1024) resolution and calibration parameters indicate that calibration was performed directly at the same resolution. However, RIS provides samples at (1920x1080) resolution with useful RGB and segmentation mask information occupying a smaller area around the middle of the frames with an aspect ratio of 4:3. Based on these observations and without knowing the exact data construction workflow of RIS, we assume that video capturing, camera calibration and labelling were performed at daVinci Xi’s resolution (1280x1024) and afterwards, the RGB and annotation images were superimposed towards the centre of (1920x1080) resolution frames. To use the provided calibration and stereo rectify the dataset we pre-process the dataset as follows. Initially, we crop the black borders in the periphery of the RGB (1920x1080) samples, which results in images smaller than (1280x1024), therefore incompatible with the calibration parameters provided. Before stereo rectifying the samples, we bi-linearly interpolate the cropped images to (1280x1024) resolution. We then de-interlace the image samples by removing all odd image rows and interpolating their values bi-linearly. Next, using OpenCV^4^ and the provided calibration parameters, we stereo-rectify both the RGB images and their corresponding tool-components segmentation masks. Finally, we get the binary tool segmentation masks by combining the rectified tool part masks. Because RIS was not designed to be used in stereo mode, the above process does not always result in frames suitable for our needs. We find that the accuracy of calibration is not consistent across all sub-datasets and also the left and the right frames were not perfectly time-synchronized. To mitigate potential issues during the multi-task training phase, we create a smaller dataset containing stereo rectified samples from sub-datasets 1,2, and 4 and we refer to it as RIS 12. The selection of those datasets was made after stereo rectifying them and visually verifying the absence of a vertical offset between corresponding pixels in the two stereo views. The time-synchronization issue between the two channels remains, however, we choose not to reject additional frames. When we evaluate the segmentation performance of our network in monocular settings we still crop, resize, and interlace the provided samples but we do not stereo rectify the reference masks and the RGB images.

#### 2) SCARED Dataset

SCARED [10] provides ground truth in form of pointclouds in the original left frame of reference. To use it with our stereo architecture we pre-process it as follows. First, using the provided stereo calibration parameters, we stereo rectify the video sequences. To generate the disparity ground truth data, for each frame, we project every reference 3D point to both the left and right stereo rectified views, measuring the horizontal displacement of the two projections in pixels, which is the disparity. Although we can generate ground truth in disparity format, we choose to only use a small subset of SCARED and also clean the provided ground truth for three main reasons. As described in the challenges’ paper, training datasets 4 and 5 have errors in the calibration parameters making them unsuitable for use in stereo setting. Furthermore, in some cases, the ground truth depth of the key-frames has outliers, such as points behind the camera or far away from the tissue surface. Lastly, the RGB video and the interpolated ground truth are not perfectly synchronized in time. To mitigate potential issues during training, we manually cleaned easily identifiable outliers in each key-frame’s ground truth. During fine-tuning, we choose only to use the key-frames of datasets 1,2,3,6,7 and not the interpolated sequences. This leaves us with 17 samples for training and 8 for evaluation a sample size similar to what was used by one of the submissions in the challenge’s paper.

## V Experiments

### A Training Phases

**Fig. 4:**
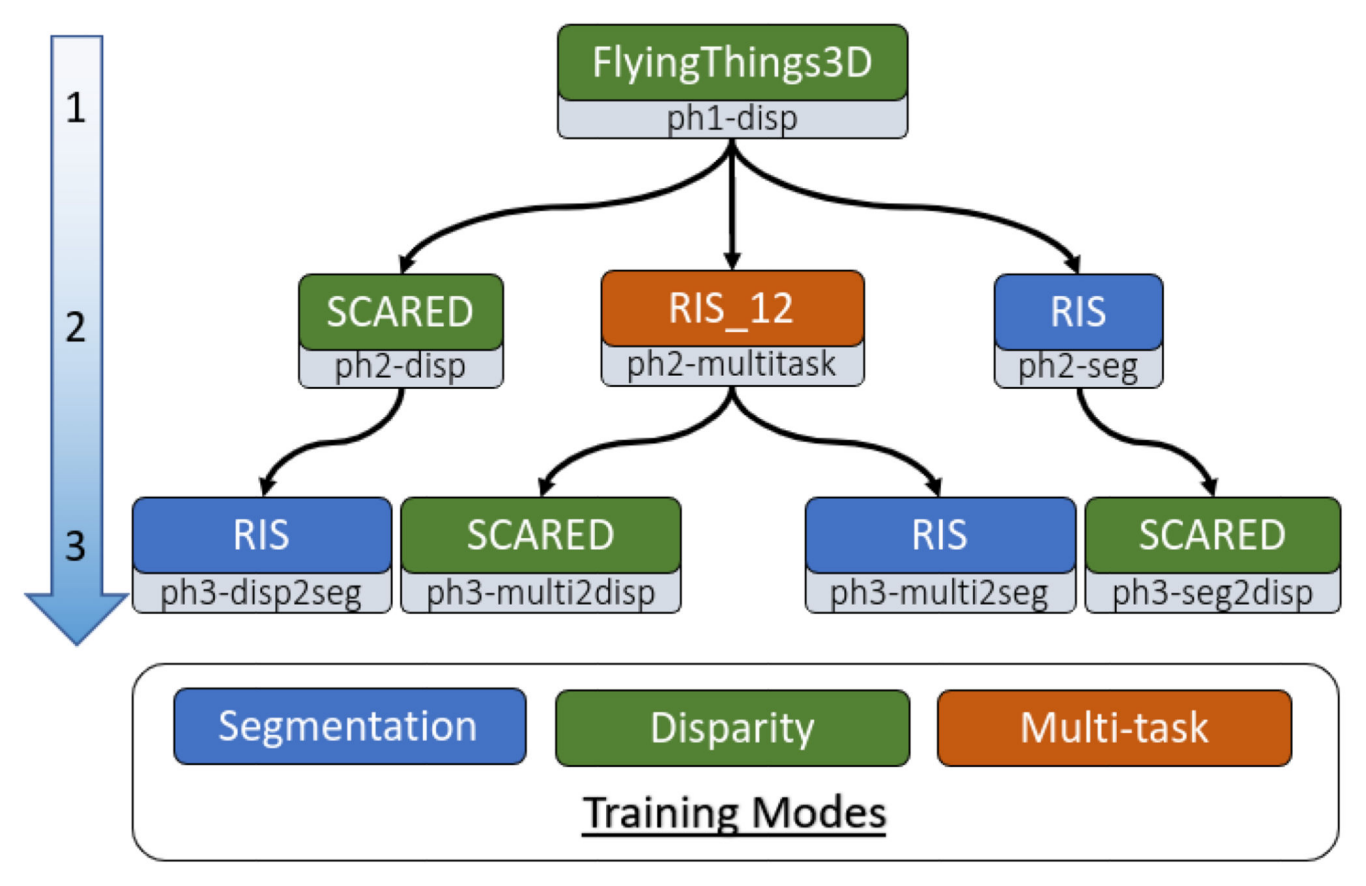
Diagram showing the training phases from (top to bottom). The 3 training modes are coloured in blue for segmentation only training, green for disparity only training, and orange for multi-task training. Each box corresponds to a different model with the upper part of the box indicating the name of the training dataset and the bottom part of the box showing the name of the model as described in section V-A. Arrows show the training sequence. Training phase 1 starts from random initialization.

To understand the benefits of multi-tasking, we train 8 versions of our model. Fig. 4 visually shows the training sequence, the datasets, and the supervision signal used during training. We train our models in phases. At each phase, we fit our model either on single-task or multi-task mode, using the appropriate dataset, and to assess the benefits of multi-tasking we evaluate both the disparity and segmentation performance. During phase 1 we pre-train only the disparity head and the shared feature encoder and use the resulting model as our baseline. In phase 2, we fine-tune our baseline model in three different settings i) jointly training both tasks, ii) only supervising disparity, and iii) only supervising segmentation. We do this to see how only supervising the segmentation head with monocular data affects the performance of the disparity head, compared to the baseline model. In addition, we investigate how accurate our model is when trained jointly on both tasks. During the last training phase, starting from the model trained jointly on both tasks, we train two additional models fine-tuning only disparity or segmentation. These experiments will show if joint training helped either task to achieve better performance compared to models trained similarly during phase 2. Furthermore, we train two additional models, starting from the phase 2 disparity and segmentation models and supervising them for segmentation and disparity respectively. The last 2 experiments are there to assess how the multi-task learning phase affects cross-task learning compared to subsequent training. We conduct the above procedure for both the ResNet34 and lightweight encoder variants, to see how the size of the encoder affects performance.

### B Training Details

All methods were implemented using PyTorch^5^(version 1.7.1), kornia^6^ and monai^7^. Training and inference scripts ran on Nvidia DGX Station V100 occupying a single 32 gigabytes (GB) GPU and using Pytorch’s Automatic Mixed Precision package(AMP). Across all experiments we used the AdamW optimizer (*β*_1_ = 0.9, *β*_2_ = 0.999) with weight decay of 1e-4.

#### 1) Pre-training

We pre-trained our disparity sub-network without the segmentation layers, fully supervised on FlyingThings3D [35] for 150 epochs and batch size of 15. We used one cycle learning rate policy [37] with a maximum learning rate of 1e-3, 1455 steps per epoch, and Pytorch’s default values for the rest of the parameters. We augment the training data using random crops of size (640x384), colour normalization based on ImageNet [38] statistics, and random vertical flips with a probability of 0.5. During this phase, we supervised the disparity across all scales *S_i_* using the smooth L1 loss (1), ignoring ground truth disparity values of over 320 pixels.

#### 2) Multi-task learning

We add the segmentation layers to the shared feature encoder, alongside the disparity head and load the baseline model from phase 1. We train the multi-task framework jointly, end-to-end, on the stereo rectified RIS [1] datasets 1 and 2 and evaluate on dataset 4, to avoid issues caused by bad rectifications as described in section IV-B.1. We train for 70 epochs with a batch size of 10 and a constant learning rate of 1e-4. We performed colour normalization based on SCARED statistics, random crops of size (640x512), and random vertical flips with a probability of 0.5. We supervise only the final output of both the segmentation and the disparity heads using the 𝓛_mt_ using *α*_mt_ = 0.2 because we experimentally found that it provides good optimization for both tasks. In (7) we set *α*_ss_ = 0.9 and *β*_ss_ = 0.7. In (5), we set $\beta =\frac{1-0.15}{0.15}$ because we calculated that tool pixels in the training dataset cover, on average,15% of the samples’ area.

#### 3) Domain fine-tuning

We Fine-tune models on a single task in both training phases 2 and 3 but each time starting from a different pre-trained version of the network. During phase 2 we start from the baseline model of phase 1 whereas in phase 3 we start from the phase 2 models. The hyperparameters are chosen to be the same across all phases for simplicity.

We fine-tune the disparity task, with a constant learning rate of 1e-4, for 600 epochs at batches of 7 samples. We only use a small part of SCARED for the reasons explained in section IV-B.2. In addition, we do not train with frames depicting tools because we found that frames in dataset 6 have shifted ground truth with respect to the RGB. We augment input images using colour normalization based on SCARED statistics, random crops of size (640x384), and random flips with a probability of 0.5. Similar to training phase 1, we supervise all intermediate disparity scales using the smooth L1 loss (1) as described in section V-B.1

We fine-tune the segmentation task on the monocular version of RIS using 𝓛_seg_ (4) and setting $\beta =\frac{1-0.15}{0.15}$ in (5). Training is performed for 40 epochs with a batch size of 16, maintaining a constant learning rate of 1e-4. Additionally, we perform colour normalization based on SCARED colour statistics.

### C Evaluation

Depending on the target dataset, we evaluate our method for depth, disparity, and segmentation. For RIS and SCARED we report the performance of our models in the same test sets used in each challenge. To quantitatively evaluate segmentation performance, we use the 10 test subsets of RIS and we report accuracy as the mean IoU across all of them. Because those frames are not stereo rectified, we run our networks in single-task mode. For depth and disparity, we evaluate our methods in the stereo rectified version of the SCARED, created through the process described in section IV-B.2. We report the final error as the mean error between datasets 8 and 9 which in turn is the mean across 4 video sequences plus a single frame. During the evaluation, we follow the recommended evaluation protocol and evaluate with ground truth frames whose annotations exceed 10% of image coverage in the original frame of reference. We normalize input samples using SCARED statistics and we assess performance based on the following evaluation metrics. Additionally, we fine-tuned two recent state-of-the-art disparity methods, CFNet [39] and Raft-Stereo [40], using their official code repositories and recommended training parameters and report performance after converting their output to the depth domain.

- Disparity Mean absolute error or one dimensional End-Point-Error (EPE) as is commonly referred to in the literature is the mean absolute error between the reference and the predicted disparity values.
- Disparity Bad3: The percentage of pixels whose estimated disparity diverges more than 3 pixels from the ground truth. This metric is harder to optimize compared to EPE.
- Depth MAE measured in mm distance.
- Segmentation Intersection-over-Union (IoU), also known as Jaccard Index is defined as the area of the intersection between the reference and the predicted segmentation masks divided by the area of their union.

### D Multi-task Training Ablation Study

To investigate how multi-task training affects accuracy compared to single-task learning, we conduct two experiments and compare their results with the multi-task models of phase 2. To eliminate larking variables, we keep all hyper-parameters and data the same as those used during multi-task training and we optimize the ph2-multitask model in the following two settings. First we train only with disparity self-supervision by setting 𝓛_seg_ = 0 in (3). Second we train only the segmentation task by setting 𝓛_ss−disp_ = 0 in (3)

### E Run-time Performance Analysis

We conduct a run-time performance analysis measuring both inference time and GPU memory allocation, to quantify the performance gains our multi-task networks yield, over their single-task counterparts running in sequence. Specifically, we test both ResNet34 and lightweight variants in: i) multi-task mode, ii) disparity only mode and iii) segmentation only mode. Additionally, we measure the time it takes for each encoder variant to infer features. For this analysis, we preload two 1280x1024x3 random tensors in GPU memory simulating RGB frames in the resolution of SCARED and RIS datasets. We then run inference 1000 times and measure the total inference time in seconds. By comparing the elapsed time, we can identify exactly how much time the shared architecture gains over its single-task counterparts running in sequence. Additionally, we enlist the GPU memory usage as reported by the nvidia-smi tool. During this experiment, networks are set to evaluation mode and run at full precision (float32). The disparity search space is set to 320 pixels for the multi-task and disparity experiments. Finally, we conduct the same analysis for other disparity and segmentation architectures following the same protocol and compare them with ours.

## VI Results and Discussion

Following the training scheme described in section. V-A and depicted in Fig.4, we end up with 16 models, 8 for each encoder variant(resnet34 and light). In order to easily distinguish between them, we introduce the following notation. We identify a model by its latest training phase and its training mode. To designate the training phase we use the phase identifier -ph#, where # corresponds to the training phase number. For phase 2 models, the training mode is specified as -disp for disparity only training, -seg for segmentation only training and -multitask for multitask training. In phase 3, the training mode and sequence are identified as -ph3-${source}2${training-mode}. Where the ${-source} indicates from which phase 2 model we start training from and ${training-mode} identifies the optimization task. In this section, we use a combination of the above descriptors to refer to a specific model, for instance, resnet34-ph3-multi2seg refers to our ResNet34 variant which is trained on synthetic data during phase 1, fine-tuned for both tasks jointly during phase 2, and lastly fine-tuned for only segmentation during phase 3. Similarly, light-ph2-seg corresponds to our lightweight variant trained on synthetic data during phase 1 and optimized only for the segmentation task during phase 2.

**Fig. 5:**
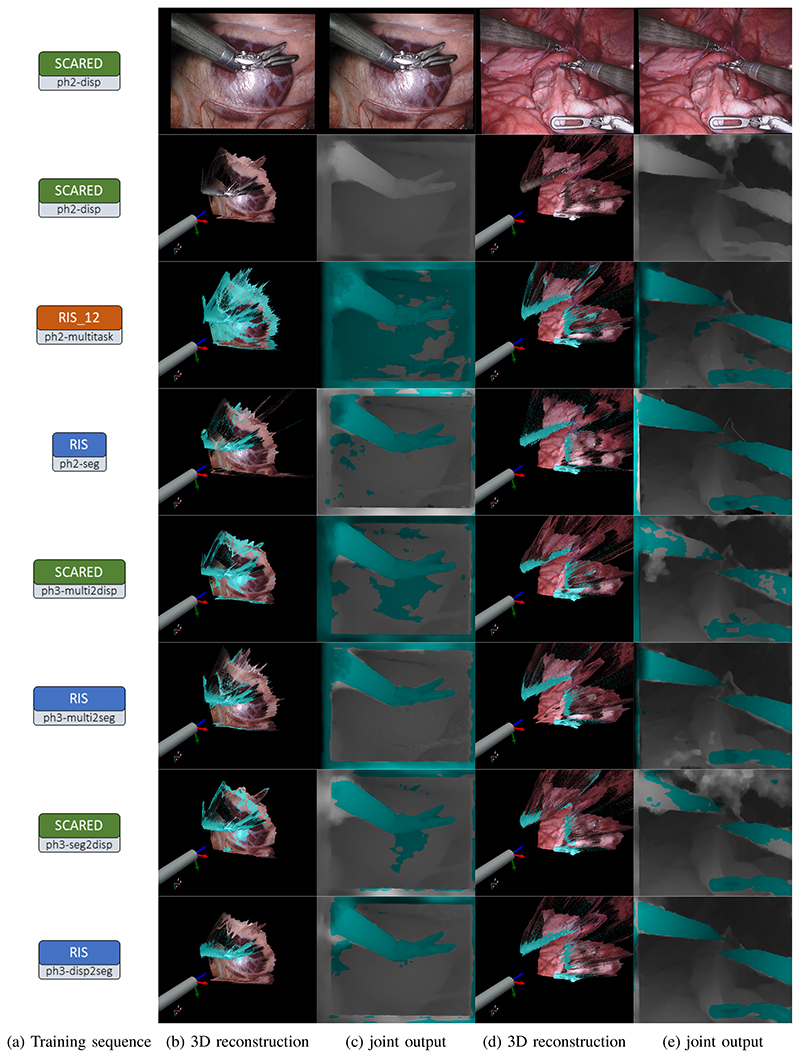
Joint disparity and segmentation prediction for a RIS (second and third column) and a SCARED (fourth and fifth column) test frame. (a) indicates the model variant used to generate each row’s output according to Fig.4. (c) and (e) show the segmentation and disparity outputs of our method, where pixels coloured in shades of cyan are classified as tools and their intensity corresponds to disparity. (b) and (d) show a third-person view of the 3D reconstructed output of our network and a model of an endoscope. ph2-disp does not predict accurate segmentation because it is not yet trained for this task. Segmentation fine-tuned models offer the best multi-task accuracy and their performance is consistent between datasets.

Fig. 5 shows qualitative results of all lightweight phase 2 and 3 models running inference with frames from both RIS and SCARED. Our network demonstrates excellent generalization capabilities, especially when trained with segmentation data(ph2-seg and ph3-multi2seg models). Table. I shows the performance of every model variant, in both tasks, across different training phases. Based on those metrics, we identify light-ph3-disp2dseg as the best overall model, achieving 89.15% mIoU on RIS and 3.18 mm depth MAE on SCARED. Our most significant finding however is the light-ph2-seg model achieving 89.08% mIoU on RIS and 3.41 mm depth MAE on SCARED without ever being exposed to surgical disparity annotated data.

### A Phase 1

When evaluating on FlyingThings3D, the resnset34-ph1-disp architecture achieves 1.37 EPE and 5.54 bad3 whereas the light-ph1-disp architecture achieved 1.29 and 6.39 respectively. Although the accuracy of both models is similar in FlyingThings3D, their performance in SCARED is vastly different, where the lightweight model achieves EPE error of about 17 pixels and the ResNet34 variant reaches EPE of over 67 pixels. We attribute this to the different capacities of the two backbones. The ResNet variant is able to fit the target domain much better but has poor performance in domain shifts compared to the network with the much smaller encoder. Phase 1 models are not able to make meaningful segmentation predictions as they are not yet trained for this task.

### B Phase 2

Overall the disparity estimation performance of the lightweight architecture in the surgical domain is better than the ResNet’s. The opposite can be said for the segmentation task. We believe that this is a result of the difference in the capacity of the two models in combination with the datasets’ size. As described in section IV-B.2, during single-task disparity training on SCARED, we use only 17 samples whereas for single-task segmentation training we use 1575 frames. During disparity only fine-tuning, the loss curves did not indicate over-fitting.

As expected, disparity only training (ph2-disp) models improve the disparity and depth estimation performance over -ph1-disp models. light-ph2-disp is our best performing disparity and depth model, achieving depth error and disparity EPE of 2.85 and 2.92 respectively. The ResNet variant demonstrates slightly worse performance reaching 4.41 EPE and 3.72 mm depth MAE, but improves compared to its pre-trained version. Because the segmentation heads of ph2-disp models have not been trained, their segmentation output is still random.

ph2-multitask models improve in disparity and depth estimation over their phase 1 models and are also able to predict segmentation outputs. The lightweight variant achieves 66.50% mIoU while the ResNet backbone model reaches 71.22%. The segmentation performance of the ph2-multitask model is not as good as the ph2-seg. As the ablation study in section VI-E suggests, this is because we perform multi-task training on RIS 12 which has significantly fewer data compared to the original dataset.

ph2-seg models, whose output can be seen in the fourth row of Fig. 5, are fine-tuned on monocular segmentation data and can reach close to state-of-the-art binary segmentation performance. The lightweight version achieves 89.08% while the ResNet34 variant reaches 90.06% mIoU in the RIS test set. Even more important is the fact that both variants vastly reduce the disparity error compared to ph1-disp models. The light-ph2-seg network achieved depth MAE of 3.41 mm and disparity EPE of 3.79 pixels. This is an error reduction of 61.73% (8.91 to 3.41) and 77.73% (17.02 to 3.79) in depth MAE and EPE metrics respectively, over the light-ph1-disp model. Similarly, the resnet34-ph2-seg variant reduced its EPE by 86.36% (67.16 to 9.16) and its depth MAE by 75.13% (25.61 to 6.37). This is the most important finding of this work because it shows that while we supervise the segmentation task with monocular data, we adapt the shared feature encoder to the target domain which leads to improvements in the disparity performance. Notably the light-ph2-seg model achieves better disparity performance compared to the ph2-multitask model. The performance difference can be attributed to the disparity self-supervision technique, used in the ph2-multitask model, not being robust to occlusions and pixel misalignment of fast-moving objects due to imperfect time-synchronized stereo channels.

### C Phase 3

#### 1) Jointly Pre-trained Models

All ph3-multi2* models improve disparity performance over ph2-multitask models. The ph3-multi2seg models also show improvement in disparity and depth over the ph2-multitask models. While the ph3-multi2seg models are not as accurate at predicting disparity and depth as the ph2-disp models, considering that during training they have never been exposed to the SCARED dataset, their performance is impressive and further prove that segmentation supervision signals are beneficial for adapting the disparity task. The ph3-multi2seg models’ depth MAE is 3.61 mm and 5.08 mm for the lightweight and the ResNet backbones respectively. Interestingly, the light-ph3-multi2seg model is overall less accurate than the light-ph2-seg model. This segmentation performance drop may be due to the limited capacity of the network (see section. VI-F) trying to serve both tasks. The disparity performance deterioration of the light-ph3-multi2seg compared to light-ph2-seg is because the former builds on top of the light-ph2-multi model that was optimized using the self-supervised disparity terms leading to decrease in performance around thin structures and occluded regions.

In contrast resnet34-ph3-multi2seg shows better over-all performance over the resnet34-ph2-seg because its bigger capacity benefits from more data during training and becomes the best segmentation model among our models.

The segmentation performance of ph3-multi2disp drops slightly compared to the ph2-multitask model by 2.32% for the lightweight variant and 7.5% for the ResNet34. This may be attributed to the absence of samples depicting tools during the ph3-multi2disp optimization phase which in turn drives the feature encoder towards producing better features for tissue areas. Although, ph3-multi2disp models are less accurate compared to the ph2-disp models by 0.12 mm for the lightweight architecture and 0.56mm for the ResNet34, the former are able to predict segmentation masks.

#### 2) Subsequently Trained Models

The ph3-disp2seg models gain segmentation capabilities that the ph2-disp models lack. The light-ph3-disp2seg achieves 3.18 mm depth MAE and 89.15% mIoU which makes it the best overall model of our study. The resnet34-ph3-disp2seg model is less accurate than the resnet34-ph3-multi2seg network and also exhibits slightly worse performance compared to the resnet34-ph2-seg model. The ph3-seg2disp models lose 22.85% and 20.46% segmentation accuracy compared to the ph2-seg models for the lightweight and ResNet variants respectively. The light-ph3-seg2disp model is about 0.1 mm less accurate compared to the light-ph2-disp model. Interestingly the resnet34-ph3-seg2disp model is the most accurate disparity version of our ResNet architecture achieving 3.03 depth mm MAE, 0.69 mm less than the resnet34-ph2-disp model. This is probably because the resnet34-ph2-disp was exposed to more domain relevant data during phase 2.

It is worth noting that the ph2-seg2disp and ph3-disp2seg cannot be directly compared with the ph2-seg and multi2seg models in terms of disparity error because the last two models have never been exposed to surgical disparity data.

### D Method Comparison

A quantitative comparison between our method and others in the literature can be found in Table. II. The best disparity model on SCARED is from the Dimitris Psychogyios 1 submission [10] using DeepPrunner, and achieving 2.33 depth MAE. The best segmentation performance on RIS had been achieved by ST-MTL [34] at 91% mIoU. Our best disparity model is the light-ph2-disp single-task disparity model which achieves 2.85 mm depth MAE and is about 0.5 mm less accurate than DeepPrunner while outperforming the winner and runner up of the SCARED challenge (two first rows of Table. II). The light-ph2-seg that has never been exposed to SCARED data, achieves 3.41 mm depth MAE and simultaneously reaches 88.39% mIoU in RIS, 2.5% less accurate compared to the current state-of-the-art. It is worth mentioning that the light-ph2-seg model, has been adapted to the surgical domain using monocular segmentation data and yet is more accurate compared to the fully supervised Trevor Zeffiro SCARED submission using a PSMNet. Our light-ph3-disp2seg model is our best overall model, reaching 89.15% mIoU on RIS and 3.18 mm depth MAE on SCARED. Finally the resnet34-ph3-disp2seg demonstrates poor disparity estimation performance but achieves 90.46% mIoU and is our most accurate segmentation model. Although our models are not able to set state-of-the-art in either dataset, they can closely compete with other approaches in the literature while being substantially faster, simultaneously predicting both disparity and segmentation, as demonstrated in section. VI-F, enabling their use in real-time applications.

### E. Multi-task Training Ablation Study

**Fig. 6:**
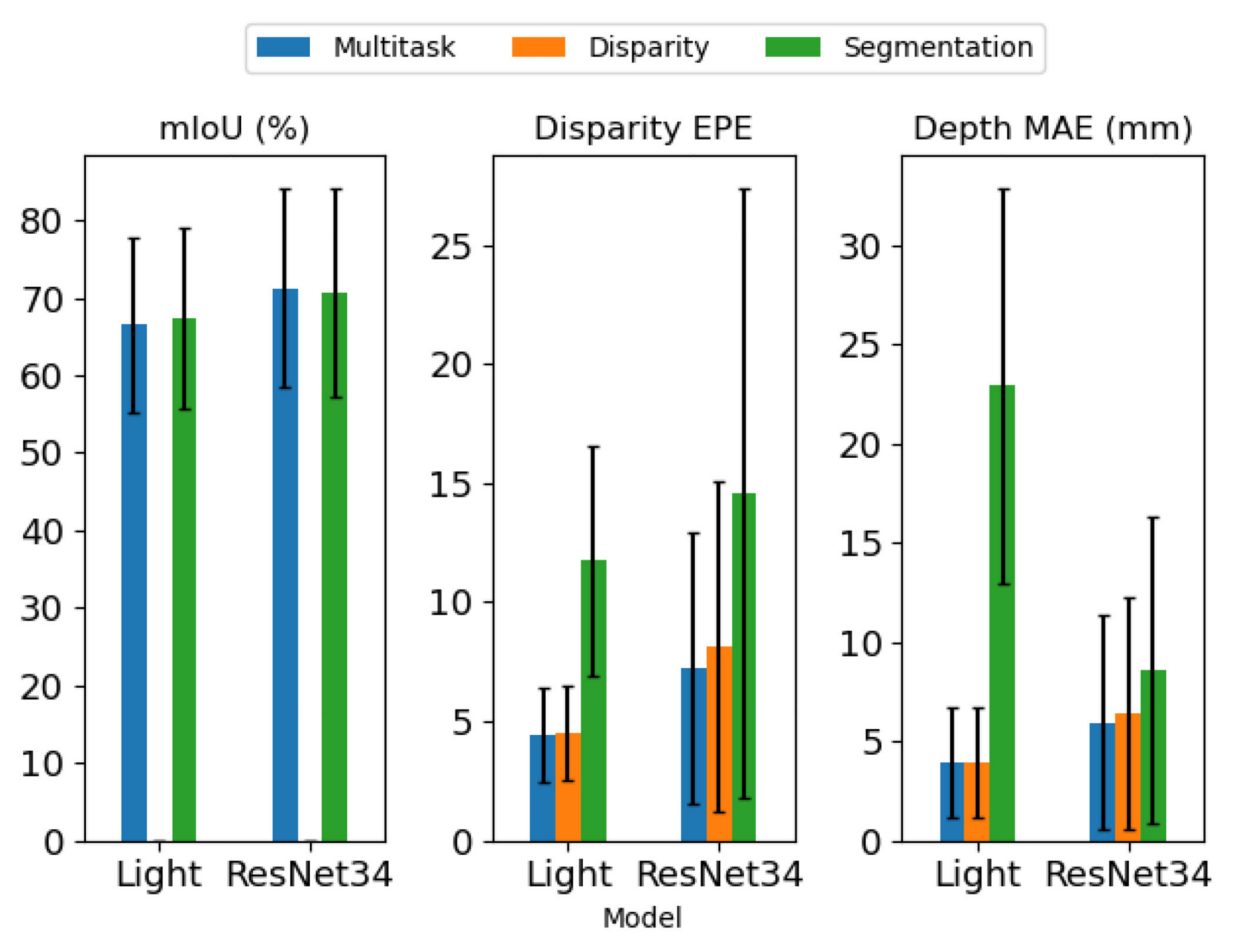
Bar graph showing the effects of multi-task learning training (blue), compared to segmentation only training (green) and self-supervised disparity training (orange) in RIS 12. On the left, segmentation accuracy in RIS, in the middle disparity EPE, and on the right depth MAE in SCARED. Among those training methods, multi-task learning achieves almost always better results. We do not include the segmentation performance of the disparity models because their output is random.

Fig.6 shows the performance of the ph2-multitask model compared to models trained only with disparity self-supervision (blue) and only with segmentation supervision (yellow). Multi-task learning achieves better accuracy compared to disparity only self-supervision in both depth and disparity estimation. For the segmentation task, the ResNet34 segmentation network is less accurate compared to its multi-task counterpart and the opposite happens in the case of the lightweight variant, nevertheless, the difference in segmentation performance between the multi-task model and the fully supervised segmentation model is marginal. Another interesting observation from the disparity and depth graphs is that ph2-multitask models achieve lower error compared to *-seg models unlike what we observed in Table. I. The only difference between this ablation study and the segmentation models in section VI-B is the use of RIS 12 instead of RIS, this tells us that the effects of the cross-task disparity adaptation scheme using monocular segmentation data scales with the amount of segmentation data used during training. Additionally, the segmentation accuracy of the segmentation-only models, of this experiment, is lower compared to the ph2-seg models because they are trained on RIS 12 instead of the full dataset. Finally, the large depth MAE error of the lightweight segmentation model is caused by disparity pixels with values very close to zero which translates to large depth values.

### Run-time Performance Analysis

Table. III shows a run-time performance comparison between the different configurations of our method and other methods from the literature. For this experiment, the disparity and segmentation models are configured to estimate only disparity and segmentation respectively, whereas multi-task models are configured to jointly predict segmentation and disparity. For our networks, we also report metrics for just inferencing features (encoder).

All network configurations of our lightweight architecture occupy less than 2GB of GPU memory facilitating their integration in low-resource hardware. The ResNet34 variants are bigger compared to the lightweight variants, both due to the encoder size and also due to the increased size of the heads to accommodate the longer feature vectors.

From the elapsed time processing 1000 samples, we see that the multi-task networks are faster compared to both their standalone counterparts running in sequence, by approximately the time it takes for their encoder to produce features. Specifically, during 1000 iterations, the lightweight architecture saves 29.66 + 9.55 − 37.23 = 2.07 seconds and ResNet34 architecture saves 54.81 + 36.71 − 79.56 = 11.96 seconds. This is because in multi-task mode the feature encoder has to run only once for the left image to serve both tasks. The feature extraction time gained from the multi-task architecture can be a deciding factor when choosing between single and multi-task networks in time-critical applications especially when a larger feature encoder is used. The run-time performance bottleneck of both multi-task networks variants is the disparity head. Our ResNet variant configured in segmentation only mode, completes inference in 36.71 seconds, about 4 times slower compared to its lightweight counterpart. The run-time performance difference is not as significant between the two disparity variants, with the ResNet disparity network inferring 1000 times at 54.81 seconds compared to the 29.66 seconds it takes for the lightweight architecture. This is because most of the inference time in the disparity head is spent during the cost volume construction phase which is the same regardless of the variant and is implemented in pure python code. Overall, in multi-task mode, our ResNet34 variant can process 1000 stereo samples in 79.56 seconds whereas our lightweight variant is around 50% faster, completing inference in just 37.23 seconds.

Comparing both architectures with others in the literature, our lightweight variant is fast and requires less than 2GB of GPU memory, even in multi-task mode. Our lightweight architecture in single-task mode is approximately an order of magnitude faster compared to other single-task approaches. In a real-world scenario, the run-time figures in Table. III will be slightly higher as data would have to be normalized and loaded to the GPU. Using Pytorch’s asynchronous data loader objects, our lightweight variant was able to predict the whole SCARED test sequence (5909 frames) averaging around 22 FPS when processing stereo 1280x1024x3 inputs in batches of 1. This translates to roughly 45 seconds for inferencing 1000 samples in multi-task mode which is 8 seconds slower compared to the reported 37.23 seconds.

## VII Conclusion

The paper has presented a novel framework for the joint estimation of disparity and surgical tool segmentation masks from stereo-endoscopic images. Starting from a pre-trained disparity network, we investigated various training schemes to incorporate segmentation and perform domain adaptation in surgical scenes. We showed that in addition to classical multi-task learning approaches that rely on stereo rectified image pairs, we were able to improve the accuracy of our disparity network by training only the segmentation head and the shared feature encoder only using monocular data. We reported results in both RIS and SCARED datasets where our best overall model achieved near state-of-the-art segmentation performance and good disparity results. We also achieve impressive disparity performance in SCARED using our cross-task adaptation scheme and monocular segmentation data. Additionally, our best overall model light-ph3-seg2disp is able to process (1280x1080) stereo input and make predictions for both tasks jointly at a very efficient 22 FPS.

In future work, we aim to leverage the modular and pyramidal design of this framework to incorporate more tasks such as scene defogging to handle surgical smoke. Further segmentation categories can also be incorporated to label organs or different instrument segments and potentially also improve the disparity performance by regularising over meaningful regions. In addition to including temporal constraints, an investigation could be conducted to assess how data augmentation affects the accuracy of models trained. Finally, the cross-task adaptation characteristic that our model exhibits can potentially be used in combination with an online adaptation mechanism similar to MAD [17] and improve adjacent tasks while partially self-supervising the disparity sub-network.

## Figures and Tables

**Table I:** Segmentation and disparity/depth performance evaluation of both Resnet34 and lightweight encoder multi-task models for every variant shown in Fig.4. Each row corresponds to a different performance metric. Bold indicates the best score for a particular metric among the different variations of our networks. We underline and overline the best performance of the lightweight and ResNet34 variant respectively

Model	ph1-disp		ph2-disp		ph2-multitask		ph2-seg		ph3-multi2disp		ph3-multi2seg		ph3-seg2disp		ph3-disp2seg	
Encoder Metric	Light	ResNet34	Light	ResNet34	Light	ResNet34	Light	ResNet34	Light	ResNet34	Light	ResNet34	Light	ResNet34	Light	ResNet34
IoU (Area%)(↑)	14.73	14.33	14.73	14.65	66.50	71.22	89.08	90.06	64.18	63.71	88.33	$\bar{90.46}$	66.22	69.60	89.15	90.14
Bad3 (pixels%)(↓)	38.76	69.98	**28.62**	35.46	33.71	38.37	31.47	40.50	29.37	41.01	33.11	36.43	28.85	$\bar{32.57}$	31.62	40.85
Depth (mm)(↓)	8.91	25.61	**2.85**	3.72	3.91	5.84	3.41	6.37	2.98	4.28	3.61	5.08	2.93	$\bar{3.03}$	3.18	6.37
EPE (pixels)(↓)	17.02	67.16	**2.92**	4.41	4.45	7.22	3.79	9.16	3.04	4.78	4.11	6.13	3.08	$\bar{3.08}$	3.46	9.89

**TABLE II:** Quantitative comparison of our best performing models with other models from the literature. We underline the best performance among all models and with bold we indicate the best performance among our models.

Model	Depth MAE (mm)(↓)	mIoU (Area%)(↑)
J.C. Rosenthal [10]	3.75	N/A
Trevor Zeffiro [10]	3.54	N/A
Dimitris Psychogyios 1 [10]	2.33	N/A
Dimitris Psychogyios 2 [10]	2.62	N/A
Sebastian Schmid [10]	2.66	N/A
CFNet [39]	2.67	N/A
RAFT-Stereo [40]	2.65	N/A
MIT [1]	N/A	88.80
UB [1]	N/A	87.50
TUM [1]	N/A	87.30
ST-MTL [34]	N/A	91.00
light-ph2-disp(Ours)	**2.85**	N/A
light-ph2-seg(Ours)	3.41	89.08
light-ph3-disp2seg(Ours)	3.18	89.15
resnet34-ph3-multi2seg(Ours)	5.08	**90.46**

**Table III:** Run-time performance comparison of our method in multi-task and single-task mode, with other disparity and segmentation learning-based approaches. We measure elapsed time as the time it takes for a network to make 1000 predictions on 1280x1024 inputs. GPU memory usage was measured using the nvidia-smi utility. DeepPrunner and HSM correspond to Dimitris Psychogyios 1 and 2 submissions of the SCARED challenge respectively. TernausNet corresponds to the MIT submission in the RIS challenge.

Model	# Parameters (Millions)	Memory usage (MB)	Elapsed time (Seconds)
lightweight encoder	0.47	1,243	2.27
lightweight disparity	2.56	1,897	29.75
lightweight segmentation	1.20	1,393	9.55
lightweight multi-task	3.30	1,933	37.23
ResNet34 encoder	21.28	1,445	12.21
ResNet34 disparity	24.17	2,455	54.81
ResNet34 segmentation	26.60	2,145	36.71
ResNet34 multi-task	29.49	2,775	79.56
CFNet [39]	23.05	10,165	567.32
RAFT-Stereo [40]	11.12	4,619	683.27
DeepPrunner [30]	7.39	4,101	321.77
HSM [41]	3.17	2,621	80.79
TernausNet [7]	25.36	3,321	110.24
ST-MTL [34]	36.62	2,773	209.63

## Footnotes

1
https://github.com/dimitrisPs/msdesis

2
https://github.com/dimitrisPs/ris2017_toolkit

3
https://github.com/dimitrisPs/scared_toolkit

5
https://pytorch.org/

6
https://kornia.github.io/

7
https://github.com/Project-MONAI/MONAI
